# Region-dependent mechanical characterization of porcine thoracic aorta with a one-to-many correspondence method to create virtual datasets using uniaxial tensile tests

**DOI:** 10.3389/fbioe.2022.937326

**Published:** 2022-10-11

**Authors:** Dongman Ryu, Seungik Baek, Jungsil Kim

**Affiliations:** ^1^ Medical Research Institute, Pusan National University, Busan, South Korea; ^2^ Department of Mechanical Engineering, Michigan State University, East Lansing, MI, United States; ^3^ Department of Convergent Biosystems Engineering, Sunchon National University, Suncheon, South Korea; ^4^ Institute of Human Harmonized Robotics, Sunchon National University, Suncheon, South Korea

**Keywords:** virtual dataset, regional variation, arterial stiffness, Fung model, holzapfel model, constrained mixture model, statistical correlations, bootstrapping

## Abstract

The simulation of the cardiovascular system and *in silico* clinical trials have garnered attention in the biomedical engineering field. Physics-based modeling is essential to associate with physical and clinical features. In physics-based constitutive modeling, the identification of the parameters and estimation of their ranges based on appropriate experiments are required. Uniaxial tests are commonly used in the field of vascular mechanics, but they have limitations in fully characterizing the regional mechanical behavior of the aorta. Therefore, this study is aimed at identifying a method to integrate constitutive models with experimental data to elucidate regional aortic behavior. To create a virtual two-dimensional dataset, a pair of uniaxial experimental datasets in the longitudinal and circumferential directions was combined using a one-to-many correspondence method such as bootstrap aggregation. The proposed approach is subsequently applied to three constitutive models, i.e., the Fung model, Holzapfel model, and constrained mixture model, to estimate the material parameters based on the four test regions of the porcine thoracic aorta. Finally, the regional difference in the mechanical behavior of the aorta, the correlation between the experimental characteristics and model parameters, and the inter-correlation of the material parameters are confirmed. This integrative approach will enhance the prediction capability of the model with respect to the regions of the aorta.

## Introduction

Recently, the simulation of the cardiovascular system and *in silico* clinical trials have garnered attention in biomedical engineering. Virtual patient cohorts and digital twins are emerging as promising strategies in precision medicine, although they are not yet fully established for clinical applications (Niederer et al., 2020; Chakshu et al., 2021). In physics-based modeling, a virtual model comprises two layers, where one layer includes a set of clinical features, and the other includes a set of model parameters. It is important to associate the model with physical or clinical features. Inadequate anatomical, physiological, and functional parameters of the heart and vasculature hinder the further development of cardiovascular devices and novel treatments (Hose et al., 2019).

The constitutive model represents the behavior of materials in the form of relationship between strain and stress. To establish physics-based modeling, an essential step is to identify the constitutive parameters and estimate their ranges based on appropriate experiments such as the tensile test of flat segments or the extension-inflation test of cylindrical segments. The mechanical properties of the blood vessel are generally determined by using a specific constitutive model. Blood vessels exhibit hyperelastic and anisotropic properties. It is well known that elastin dominates the initial linear behavior in the low-stiffness regime, whereas collagen is recruited in the high-stiffness regime (Harkness et al., 1957). To identify the anisotropic behavior of blood vessels, pairs of experimental datasets in two loading directions for the same sample are required. Biaxial testing is more reliable in determining the material parameters in the constitutive models, while uniaxial testing is advantageous in investigating the regional variation in the heterogeneous properties of the blood vessel. We previously demonstrated that the posterior side of the porcine thoracic aorta is significantly stiffer than the anterior side in the extension-inflation test, and that the longitudinal difference in the aortic mechanical properties is dependent on the circumferential region (Kim and Baek, 2011; Kim et al., 2013). Therefore, the uniaxial tensile test must be revisited in terms of the spatial description of arterial heterogeneity and its potential application to *in silico* studies of the virtual aorta.

To elucidate the regional variation of blood vessels, this study was conducted to identify a new approach to integrate a constitutive model and uniaxial test data. To this end, uniaxial tensile tests were performed on the eight sets of aortic segments in consideration of the test regions and loading directions. For an integrated prediction tool based on experimental data, we propose a three-step approach to describe the regional and nonlinear anisotropic mechanical behavior, as well as the relationship between the physical characteristics (arterial stiffness) and constitutive model parameters. First, each virtual two-dimensional (2D) dataset is constructed using a pair of uniaxial tests in the longitudinal and circumferential loading directions for the same sample, followed by the estimation of a set of anisotropic constitutive model parameters. Consequently, a number of virtual datasets are generated *via* bootstrap aggregation, which can be used to improve the predictive performance of the model. Second, the data populated using the models allows the analysis of their regional variabilities and differences in the mechanical behavior of the aorta through various statistical tools. Finally, the correlations between the arterial physical characteristics and model parameters, as well as the correlations among the model parameters are determined. The proposed approach is evaluated using three constitutive models: the Fung model, Holzapfel model, and constrained mixture model (CMM).

## Methods

**FIGURE 1:**
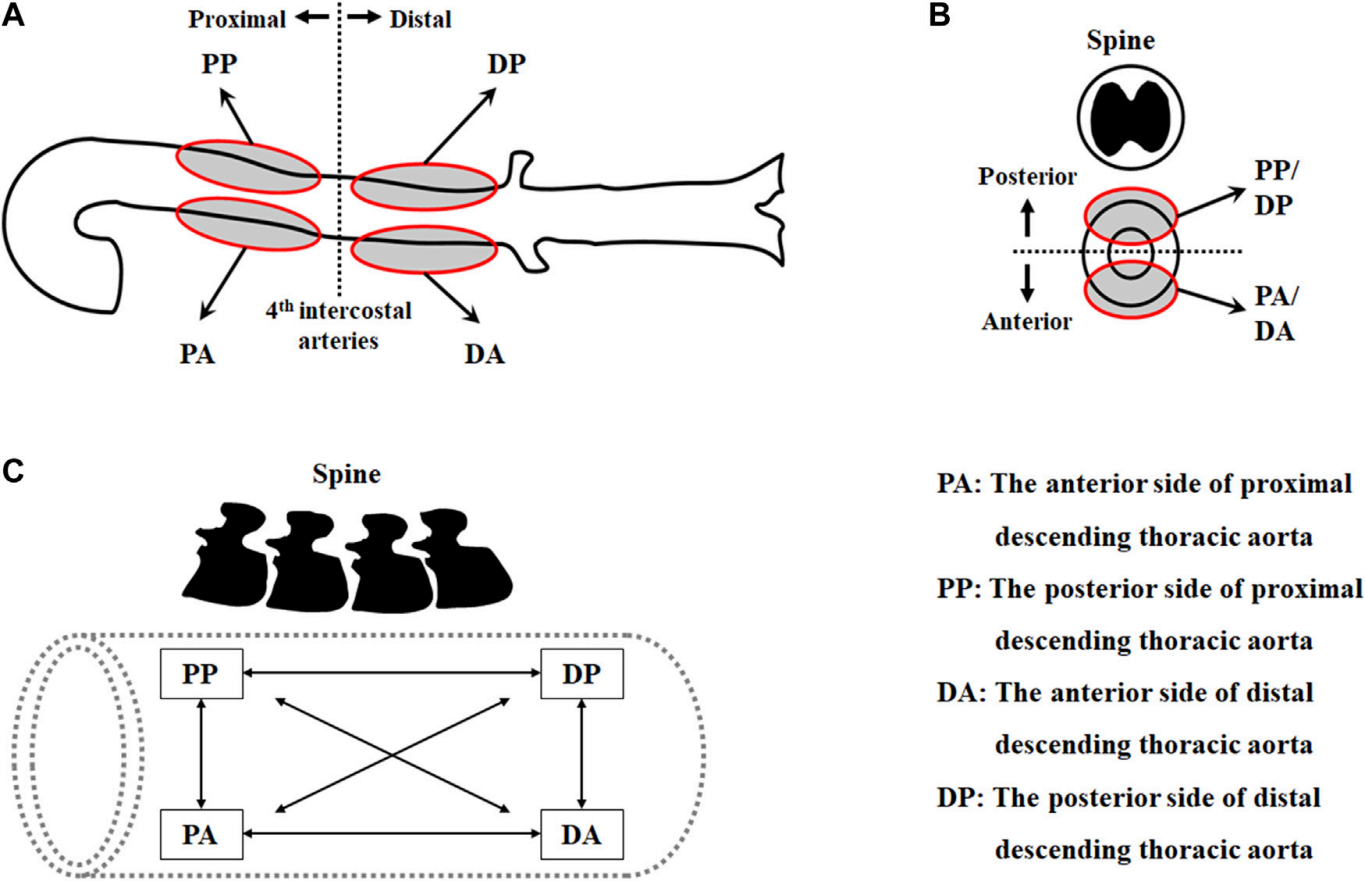
Test regions of the thoracic aorta in **(A)** the longitudinal direction and **(B)** the circumferential direction, and **(C)** pairs of datasets in two loading directions.

To investigate the regional variations in the mechanical behavior and material parameters of the aorta, the thoracic aorta was segmented into eight sets according to the circumferential direction (i.e., anterior vs. posterior), longitudinal direction (i.e., proximal vs. distal), and loading directions (i.e., circumferential vs. longitudinal) in the experiment (Figure 1), as follows:

1) circumferential specimen of the anterior side of proximal descending thoracic aorta (PAC)
2) longitudinal specimen of the anterior side of proximal descending thoracic aorta (PAL)
3) circumferential specimen of the anterior side of distal descending thoracic aorta (DAC)
4) longitudinal specimen of the anterior side of distal descending thoracic aorta (DAL)
5) circumferential specimen of the posterior side of proximal descending thoracic aorta (PPC)
6) longitudinal specimen of the posterior side of proximal descending thoracic aorta (PPL)
7) circumferential specimen of the posterior side of distal descending thoracic aorta (DPC)
8) longitudinal specimen of the posterior side of distal descending thoracic aorta (DPL).

### Sample and uniaxial tensile test

Eight fresh descending thoracic aortas from approximately 6-month-old pigs were obtained from a local slaughterhouse. They were immediately used after their loose connective tissues were removed from the adventitia. The aorta was cut in the order of circumferential and longitudinal directions with a width of 6 mm along the aortic tree. The total number of aortic segments used in this study was 35, 29, 26, 28, 25, 25, 28, and 28 for the PAC, PAL, DAC, DAL, PPC, PPL, DPC, and DPL, respectively. The thickness of the sample was measured twice at three different points using a Vernier caliper. The ends of the sample were attached to sandpaper with cyanoacrylate to avoid slipping during the experiments. Two microspheres with a 10 mm gap were attached to the center of the sample. Subsequently, the sample was mounted on the experimental device at a length of 20 mm between the clamps of the device, which was originally developed for the extension-inflation test (Kim and Baek, 2011) but was modified for the uniaxial tensile test. Before commencing the tensile test, the distance between two clamps was adjusted to flatten the sample in the absence of bending, which was confirmed by the microspheres attached to the sample. The sample was maintained in the moist state and then stretched at a speed of 2.9 mm/s until rupture. During the test, the axial force exerted on the sample was measured continuously at a sampling rate of 100 Hz with a 50 N load cell. The images of the sample were also taken using a CCD camera, and the position of the microspheres were tracked through the post imaging process with custom-written Matlab codes and were used to determine the stretch.

### Calculation of slope in stress–stretch curve

The experimental data up to fiber breakage points in which the stress–stretch curve deviated from the high-stiffness regime was used for analysis. Two distinct regimes were observed in the stress–stretch curve: A moderate gradient slope (low-stiffness) regime and a steep gradient slope (high-stiffness) regime. To quantitatively characterize the nonlinear behavior of the aortic tissues, the slope of the tangent to the stress–stretch curve was determined. The slope was determined in two steps for each regime to represent the stiffness of the aortic tissue. Since the stress-stretch curve can be approximated as a piecewise linear function, the optimal range of each linear fit before and after transition was first determined based on the coefficient of determination (*R* ^2^). The first point of the stress-stretch curve served as a starting point for the optimal range in the low-stiffness regime, and the last point of the curve was used as an endpoint in the high-stiffness regime. Next, the fitting equation to describe the stress-stretch curve was defined and the derivative of the fitting equation was determined at one point within the optimal range for each regime.

### Constitutive models for hyperelasticity

#### Stress tensor and incompressibility

A majority of constitutive models consider the elastic behavior of blood vessels to be hyperelastic. Aortic tissues are typically characterized by the hyperelastic strain energy function. The aortic wall is assumed to be an incompressible material, i.e., *J* ^2^ = det **C** = 1, where *J* is the Jacobian, and **C** is the right Cauchy–Green deformation tensor. The Cauchy stress tensor, **T**, which describes the true stress state in the material configuration, can be defined by the hyperelastic strain energy as follows:

$$T=-pI+2F\frac{\partial W}{\partial C}F^{T},$$ (1)

where *p* is the Lagrange multiplier that can be determined by the boundary conditions, **F** the deformation gradient, and *W* the strain energy function. For the uniaxial test, the deformation gradient of the incompressible material is expressed as

$$F=\left[\begin{array}{ccc} \lambda & 0 & 0\\ 0 & \lambda^{-\frac{1}{2}} & 0\\ 0 & 0 & \lambda^{-\frac{1}{2}} \end{array}\right],$$ (2)

assuming that stress is applied along the loading direction (Martins et al., 2006; Annaidh et al., 2012; de Rooij and Kuhl, 2016), where *λ* is the stretch ratio.

#### Fung model

Chuong and Fung (Chuong and Fung, 1983; Zulliger et al., 2004) proposed a strain energy function to describe the mechanical behavior of an artery under internal pressure or stretching. They considered the anisotropic property and nonlinearity of the artery, and the mechanical behavior is described through the strain energy function *W*.

$$W=\frac{c_{1}}{2}\left[\exp\left(Q\right)-1\right]$$ (3)

where

$$Q=b_{1}E_{11}^{2}+b_{2}E_{22}^{2}+b_{3}E_{33}^{2}+2b_{4}E_{11}E_{22}+2b_{5}E_{11}E_{33}+2b_{6}E_{22}E_{33}$$ (4)

$$E=\frac{1}{2}\left(C-I\right),$$ (5)

where **E** is the Green–Lagrange strain tensor, *c*_1_ is an elastic constant, and *b*_1_–*b*_6_ are parameters describing the contribution of the principal strains. The uniaxial stress–stretch relationship for the Fung model, based on Eqs 3, 5, is expressed as (by solving for the Lagrange multiplier *p* using the free boundary condition in each uniaxial loading direction):

$$T_{1}=\frac{1}{2}c_{1}\lambda^{2}\left[\exp\left(Q^{\prime }\right)\right]\left[b_{1}\left(\lambda^{2}-1\right)+b_{4}\left(\lambda^{-1}-1\right)+b_{5}\left(\lambda^{-1}-1\right)\right]-p^{\prime }$$ (6)

where

$$Q^{\prime }=\frac{1}{4}b_{1}{\left(\lambda^{2}-1\right)}^{2}+\frac{1}{4}b_{2}{\left(\lambda^{-1}-1\right)}^{2}+\frac{1}{4}b_{3}{\left(\lambda^{-1}-1\right)}^{2}+\frac{1}{2}b_{4}\left(\lambda^{2}-1\right)\left(\lambda^{-1}-1\right)$$

$$+\frac{1}{2}b_{5}\left(\lambda^{-1}-1\right)\left(\lambda^{2}-1\right)+\frac{1}{2}b_{6}{\left(\lambda^{-1}-1\right)}^{2},$$ (7)

$$p^{\prime }=\frac{1}{2}c_{1}\lambda^{-1}\left[\exp\left(Q^{\prime }\right)\right]\left[b_{3}\left(\lambda^{-1}-1\right)+b_{5}\left(\lambda^{2}-1\right)+b_{6}\left(\lambda^{-1}-1\right)\right],$$ (8)

$$T_{2}=\frac{1}{2}c_{1}\lambda^{2}\left[\exp\left(Q^{\prime \prime }\right)\right]\left[b_{2}\left(\lambda^{2}-1\right)+b_{4}\left(\lambda^{-1}-1\right)+b_{6}\left(\lambda^{-1}-1\right)\right]-p^{\prime \prime }$$ (9)

where

$$Q^{\prime \prime }=\frac{1}{4}b_{1}{\left(\lambda^{-1}-1\right)}^{2}+\frac{1}{4}b_{2}{\left(\lambda^{2}-1\right)}^{2}+\frac{1}{4}b_{3}{\left(\lambda^{-1}-1\right)}^{2}+\frac{1}{2}b_{4}\left(\lambda^{-1}-1\right)\left(\lambda^{2}-1\right)$$

$$+\frac{1}{2}b_{5}{\left(\lambda^{-1}-1\right)}^{2}+\frac{1}{2}b_{6}\left(\lambda^{2}-1\right)\left(\lambda^{-1}-1\right),$$ (10)

$$p^{\prime \prime }=\frac{1}{2}c_{1}\lambda^{-1}\left[\exp\left(Q^{\prime \prime }\right)\right]\left[b_{3}\left(\lambda^{-1}-1\right)+b_{5}\left(\lambda^{-1}-1\right)+b_{6}\left(\lambda^{2}-1\right)\right].$$ (11)

#### Holzapfel model

Holzapfel et al. (Holzapfel et al., 2000) proposed a strain energy function that includes two families of collagen fibers. They assumed that each layer of the arterial wall demonstrated similar mechanical characteristics (Schroeder et al., 2018). In this model, the strain energy function is divided into two: *W*_iso_ associated with isotropic deformations and *W*_aniso_ associated with anisotropic deformations. The neo-Hookean model is used in the isotropic response component, as follows (Ogden, 1997):

$$W_{iso}=\frac{c}{2}\left(I_{1}-3\right),$$ (12)

where *c* > 0 is a stress-like material parameter. The strain energy function associated with anisotropic deformation is proposed to be an exponential function, as follows:

$$W_{aniso}=\frac{k_{1}}{2k_{2}}\underset{i=4,6}{\sum }\left\{\exp\left[k_{2}{\left(I_{i}-1\right)}^{2}\right]-1\right\},$$ (13)

where *k*_1_ > 0 is a stress-like material parameter, and *k*_2_ > 0 is a dimensionless parameter. The fiber direction can be described as a model parameter using *I*_*4*_ and *I*_*6*_. The uniaxial stress–stretch relationship in each loading direction for the Holzapfel model based on Eq. 12 and Eq. 13 is expressed as follows:

$$T_{1}=-c\lambda^{-1}+c\lambda^{2}+\underset{i=4,6}{\sum }2k_{1}\left(I_{i}^{\prime }-1\right)\left\{\exp\left(k_{2}{\left(I_{i}^{\prime }-1\right)}^{2}\right)\right\}\lambda^{2}\cos^{2}\gamma ,$$ (14)

where

$$I_{4}^{\prime }=I_{6}^{\prime }=\lambda^{2}\cos^{2}\gamma +\lambda^{-1}\sin^{2}\gamma ,$$ (15)

$$T_{2}=-c\lambda^{-1}+c\lambda^{2}+\underset{i=4,6}{\sum }2k_{1}\left(I_{i}^{\prime \prime }-1\right)\left\{\exp\left(k_{2}{\left(I_{i}^{\prime \prime }-1\right)}^{2}\right)\right\}\lambda^{2}\sin^{2}\gamma ,$$ (16)

where

$$I_{4}^{\prime \prime }=I_{6}^{\prime \prime }=\lambda^{-1}\cos^{2}\gamma +\lambda^{2}\sin^{2}\gamma .$$ (17)

In Eqs 14–17, γ is the angle of fiber orientation measured from the longitudinal direction.

#### CMM

The CMM proposed by Humphrey and Rajagopal (2002) presents general concepts for the modeling of biological tissues. The basic principle of the model is that living tissues have a preferred state. When the preferred state is changed, the rates of production and removal and the natural configurations of multiple constituents can be changed to restore them to the preferred state. Typically, the CMM is utilized in the computational simulation of vascular growth and remodeling; however, in this study, the CMM is presented as a constitutive model that describes the arterial mechanical behavior. In addition, in the CMM, the tissue is considered to be a homogenized mixture and the deformation of the mixture as a whole is constrained by incompressibility. In the model, the stored energy functions are given by

$$W=\rho^{e}\Psi^{e}\left(C_{n}^{e}\right)+\underset{k=1}{\sum }\rho^{k}\Psi^{k}\left(\lambda_{n}^{k}\right),$$ (18)

where

$$\Psi^{e}\left(C_{n}^{e}\right)=\frac{c_{1}}{2}\left\{C_{n\left[11\right]}^{e}+C_{n\left[22\right]}^{e}+\frac{1}{C_{n\left[11\right]}^{e}C_{n\left[22\right]}^{e}-{C_{n\left[12\right]}^{e}}^{2}}\right\},$$ (19)

$$\Psi^{k}\left(\lambda_{n}^{k}\right)=\frac{c_{2}^{k}}{4c_{3}^{k}}\left\{\exp\left[c_{3}^{k}{\left({\lambda_{n}^{k}}^{2}-1\right)}^{2}\right]-1\right\}$$ (20)

for elastin and collagen fiber families, respectively. Here, $\rho^{e}$ and $\rho^{k}$ are the density of elastin and collagen fiber *k* = 1, …, 4, and $c_{1}$ , $c_{2}^{k}$ , and $c_{3}^{k}$ are intrinsic material parameters. $C_{n\left[ij\right]}^{e}$ is a component of $C_{n}^{e}={\left[F_{n}^{e}\right]}^{T}F_{n}^{e}$ . In CMM, orientations of collagen fiber families are assumed to be symmetric with respect to the axial direction. The stretches of the *k*th collagen fiber families from their natural configurations to the current configuration are calculated as described by Zeinali-Davarani et al. (2011) and Seyedsalehi et al. (2015) as follows:

$$\lambda_{n}^{k}=G_{h}^{c}\lambda^{k},$$ (21)

where

$$\lambda^{k}=\sqrt{FM^{k}∙FM^{k}},$$ (22)

$$M^{k}=\frac{F^{-1}m^{k}}{\left|F^{-1}m^{k}\right|},$$ (23)

where $G_{h}^{c}$ is the pre-stretch of the collagen, and $\lambda^{k}$ is the stretch of the fiber from the reference to the current configuration. The unit vector $M^{k}$ in the reference configuration that corresponds to $m^{k}$ is given by Eq. 23, and the $m^{k}$ is the unit vector in the direction of the k -th collagen fiber. The angle between $m^{k}$ and the first principal direction is denoted by $\alpha^{k}$ . The homeostatic stretch tensor of elastin, $G^{e}$ , which is the mapping from the reference configuration to the current configuration, is written as

$$G^{e}=diag\left\{G_{1},\;G_{2}\right\},$$ (24)

where $G_{1}$ and $G_{2}$ are the elastin pre-stretches in the circumferential and longitudinal directions, respectively. The deformation gradient of the elastin mapping from its natural state to the current configuration is $F_{n}^{e}=FG^{e}$ .

The uniaxial stress–stretch relationship in each loading direction is expressed as

$$T_{1}=-c_{1}G_{1}^{-1}\lambda^{-1}+c_{1}G_{1}^{2}\lambda^{2}+\lambda^{2}\overset{2}{\underset{k=1}{\sum }}c_{2}^{\left(k\right)}\left({\left(\lambda_{n}^{\left(k\right)}\right)}^{2}-1\right)$$

$$\times \left\{\exp\left[c_{3}^{\left(k\right)}{\left({\left(\lambda_{n}^{\left(k\right)}\right)}^{2}-1\right)}^{2}\right]\right\}{\left(\frac{G_{h}^{c}}{\lambda_{n}^{k}}\right)}^{2}\lambda^{2}\cos^{2}\alpha ,$$ (25)

$$T_{2}=-c_{1}G_{2}^{-1}\lambda^{-1}+c_{1}G_{2}^{2}\lambda^{2}+\lambda^{2}\overset{2}{\underset{k=1}{\sum }}c_{2}^{\left(k\right)}\left({\left(\lambda_{n}^{\left(k\right)}\right)}^{2}-1\right)$$

$$\times \left\{\exp\left[c_{3}^{\left(k\right)}{\left({\left(\lambda_{n}^{\left(k\right)}\right)}^{2}-1\right)}^{2}\right]\right\}{\left(\frac{G_{h}^{c}}{\lambda_{n}^{k}}\right)}^{2}\lambda^{2}\sin^{2}\alpha ,$$ (26)

where α is the angle of fiber orientation from the axial direction.

### Calculation of material parameters

The biaxial tensile or inflation test can be used to investigate the anisotropic behavior of the aorta; however, it is limited to the characterization of spatially heterogeneous material parameters in local regions. The uniaxial tensile test allows the intrinsic material characteristics in the local regions of the blood vessel to be estimated. However, it is difficult to estimate all parameters simultaneously, particularly when numerous parameters are involved. Thus, a novel data-oriented approach, i.e., the bootstrap approach, was employed in this study. More specifically, cross-mapping involving one circumferential-to-many longitudinal mapping (or vice versa) was performed to generate a virtual biaxial dataset using custom-written Matlab codes. For a virtual dataset, a set of material parameters in the region of interest was estimated individually for each loading direction. They were then integrated to determine the common range of the material parameters for the biaxial behavior in a given region. However, a single set of material parameters for the biaxial behavior may result in a large error, if the ranges of the material parameters for each loading direction are too wide or the common ranges are too narrow such as in transition. To minimize the error, the error tolerance for the iterative solution in the Matlab codes was adjusted based on the goodness of the curve fit.

### Statistical analyses

In this study, two separate statistical analyses were performed. Analysis of variance was used to identify regional differences in the experimental results, followed by the Bonferroni correction as a post-hoc test. Additionally, the Student’s t-test was performed to examine the significance of the regional differences as well as the relationship between the material parameters and experimental results.

**FIGURE 2:**
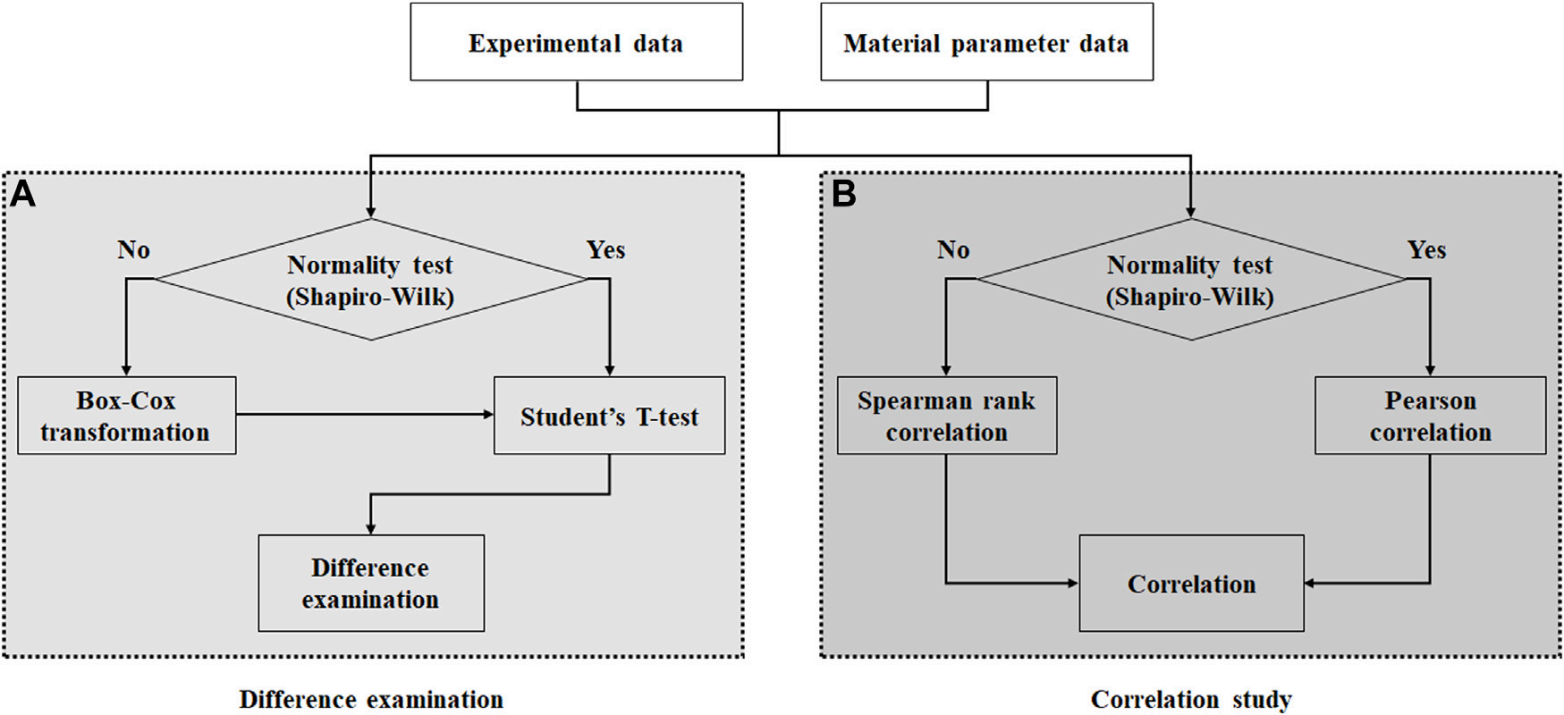
Flow chart showing procedures for **(A)** difference examination and **(B)** correlation study.

To determine the appropriate method for performing the Student’s t-test and correlation study, the following procedures were performed: 1) The normal distribution of the calculated material parameters and stiffness as well as the experimental data were examined using the Shapiro–Wilk test, as shown in Figure 2. 2) In the normality test, a significant difference was determined based on the *p*-value of 0.05. 3) The Student’s t-test was conducted to examine the differences between the material parameters based on the test regions. 4) If the normality test of the data fails, then the data can be transformed to the normally distributed data *via* Box–Cox transformations with an optimal Lambda using MATLAB codes, followed by the Student’s t-test. In addition, Pearson and Spearman rank correlation studies were conducted between the estimated material parameters and experimental data, as well as a correlation study among material parameters based on the test regions. Once the correlation coefficients (r) were calculated, the relationship strengthened in the positive and negative directions as the correlation coefficients approached +1 and −1, respectively. In addition, no correlation was observed when the correlation coefficient was 0 or the *p*-value was 0.05 or more. The SPSS software was used for the statistical analysis.

## Results

### Stress–stretch response

**FIGURE 3:**
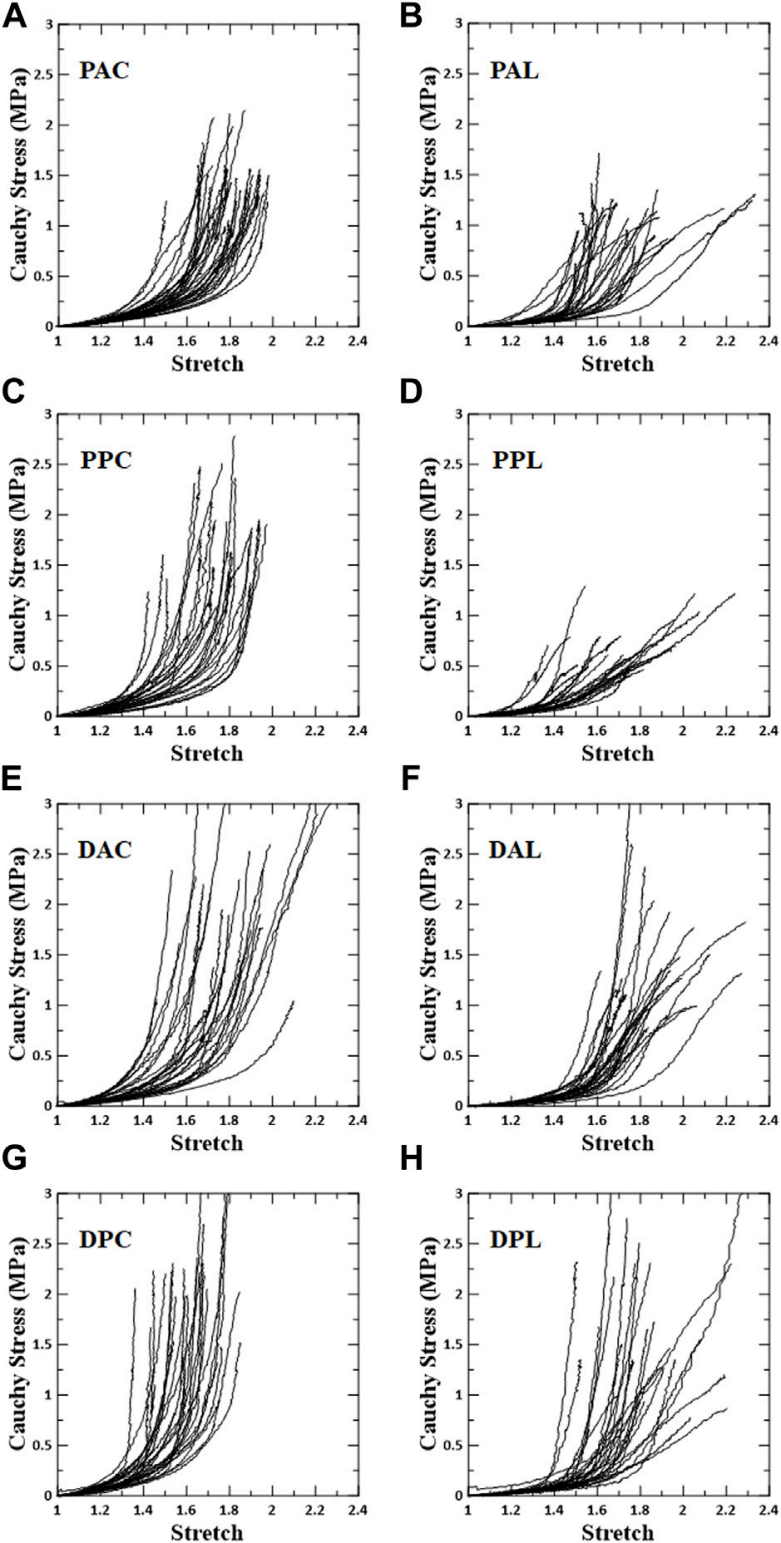
Stress–stretch response for each test region. Left column shows circumferential specimens [**(A)** PAC, **(C)** PPC, **(E)** DAC, and **(G)** DPC], and right column shows longitudinal specimens [**(B)** PAL, **(D)** PPL, **(F)** DAL, and **(H)** DPL].

**FIGURE 4:**
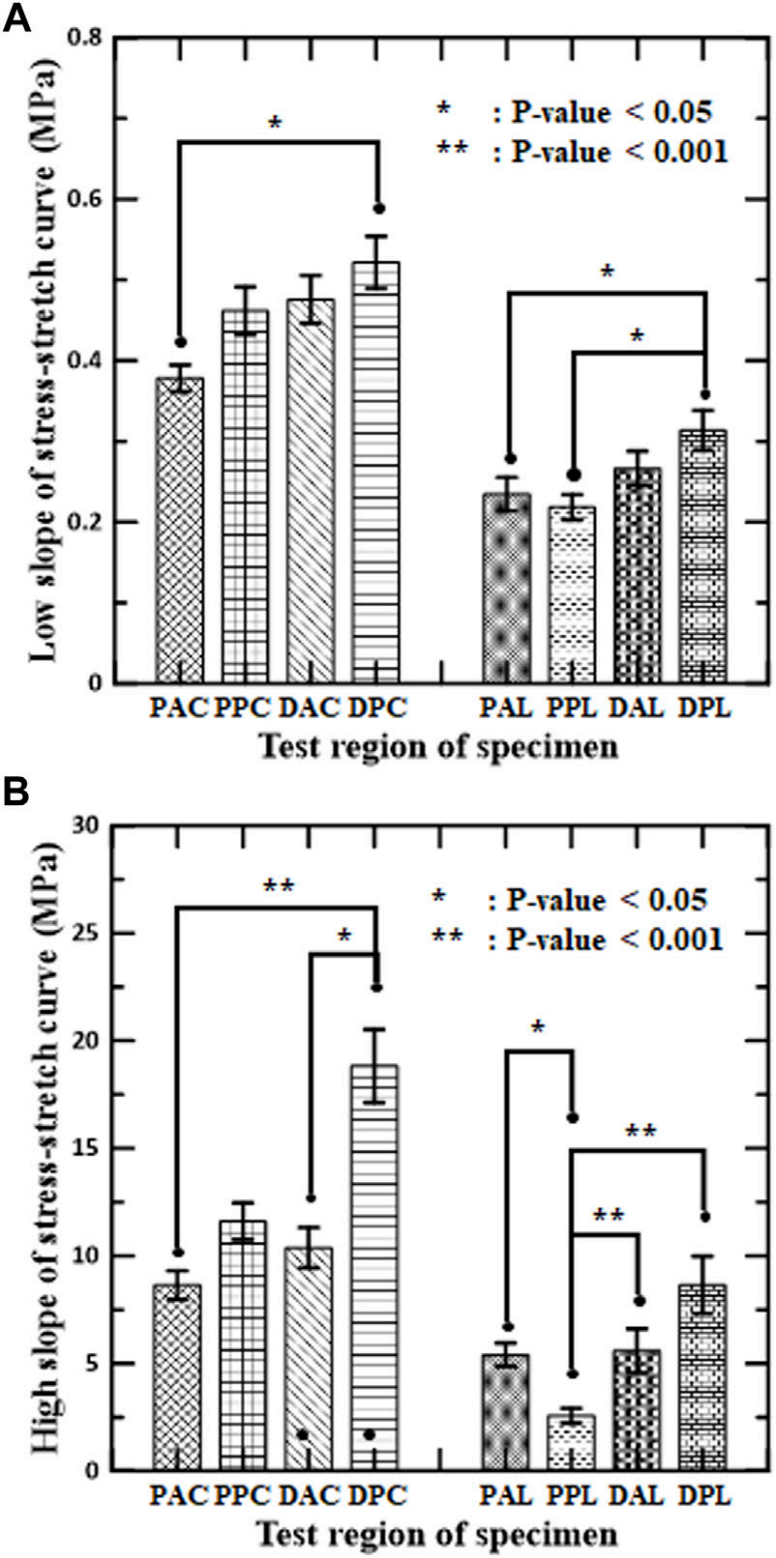
Mean and standard error of mean for slopes of stress–stretch curves of aortic tissues in **(A)** low-stiffness regimes and **(B)** high-stiffness regimes for circumferential and longitudinal specimens, respectively.

The overall stress–stretch responses were nonlinear with pronounced regional differences (Figures 3, 4). In terms of the anisotropy of the blood vessel, the circumferential specimens were stiffer than the longitudinal specimens. In addition, the transition of the circumferential specimens from the low- to high-stiffness regimes was greater than that of the longitudinal specimens.

The slopes of the stress-stretch curves for aortic tissues represent the stiffness of the aortas, which depend on the test region, the loading direction, and the regime of nonlinear behavior. The slope of the distal posterior region (DPC and DPL) was the greatest in both regimes for each loading direction. Significant difference between the anterior and posterior sides was found in the high-stiffness regime (DPC vs. DAC, and PPL vs. PAL). Significant difference between the proximal and distal regions was found at the posterior side, but only in the longitudinal specimens of both regimes (PPL vs. DPL). In addition, the interaction between the circumferential and longitudinal regions was significant for the circumferential specimens (PAC vs. DPC) in both regimes and for the longitudinal specimens (PPL vs. DPL) in the high-stiffness regime.

### Material parameters

**FIGURE 5:**
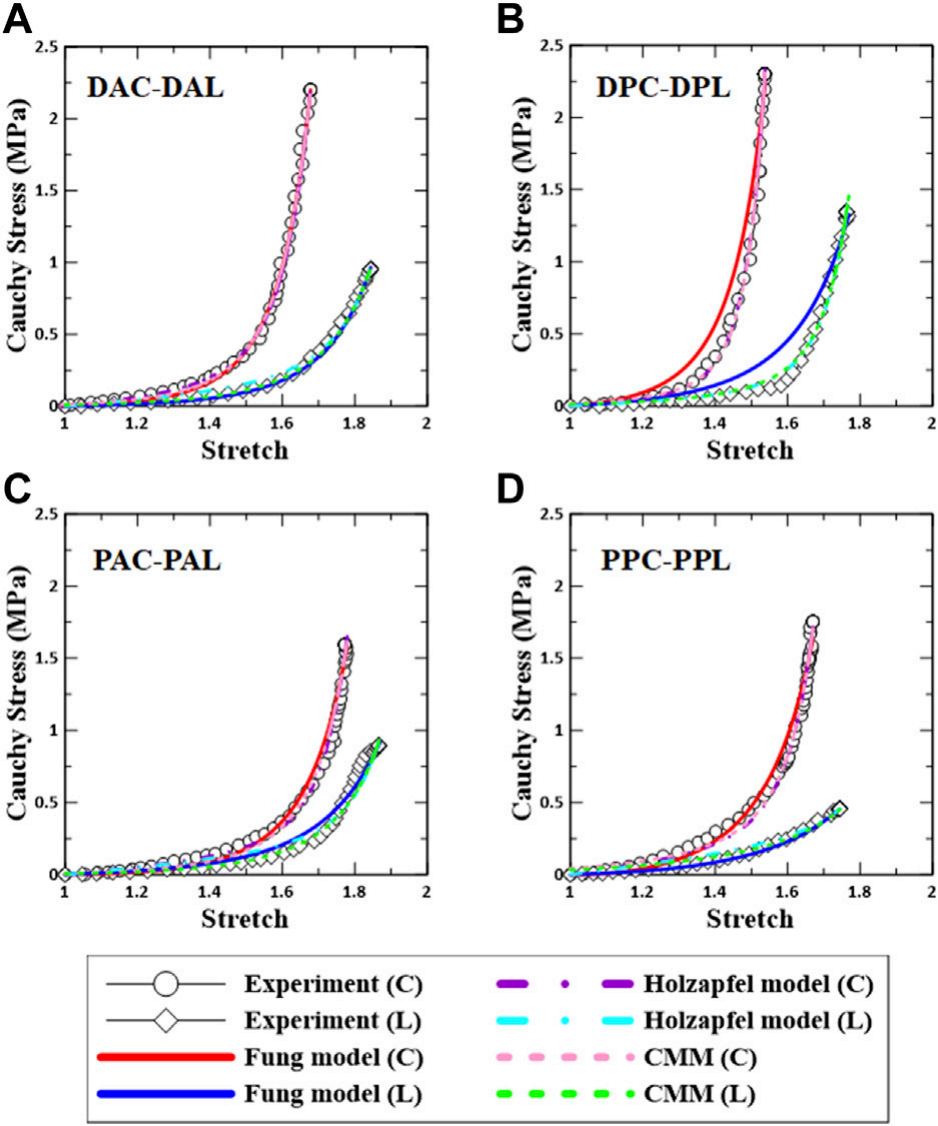
Comparison between experimental data and constitutive models based on regional aortic specimens. **(A)** DAC–DAL, **(B)** DPC–DPL, **(C)** PAC–PAL, and **(D)** PPC–PPL. C: circumferential specimen; L: longitudinal specimen.

The representative fitting results for each constitutive model along with the experimental data are shown in Figure 5. The results indicate that the fitting method used in this study is reliable.

**FIGURE 6:**
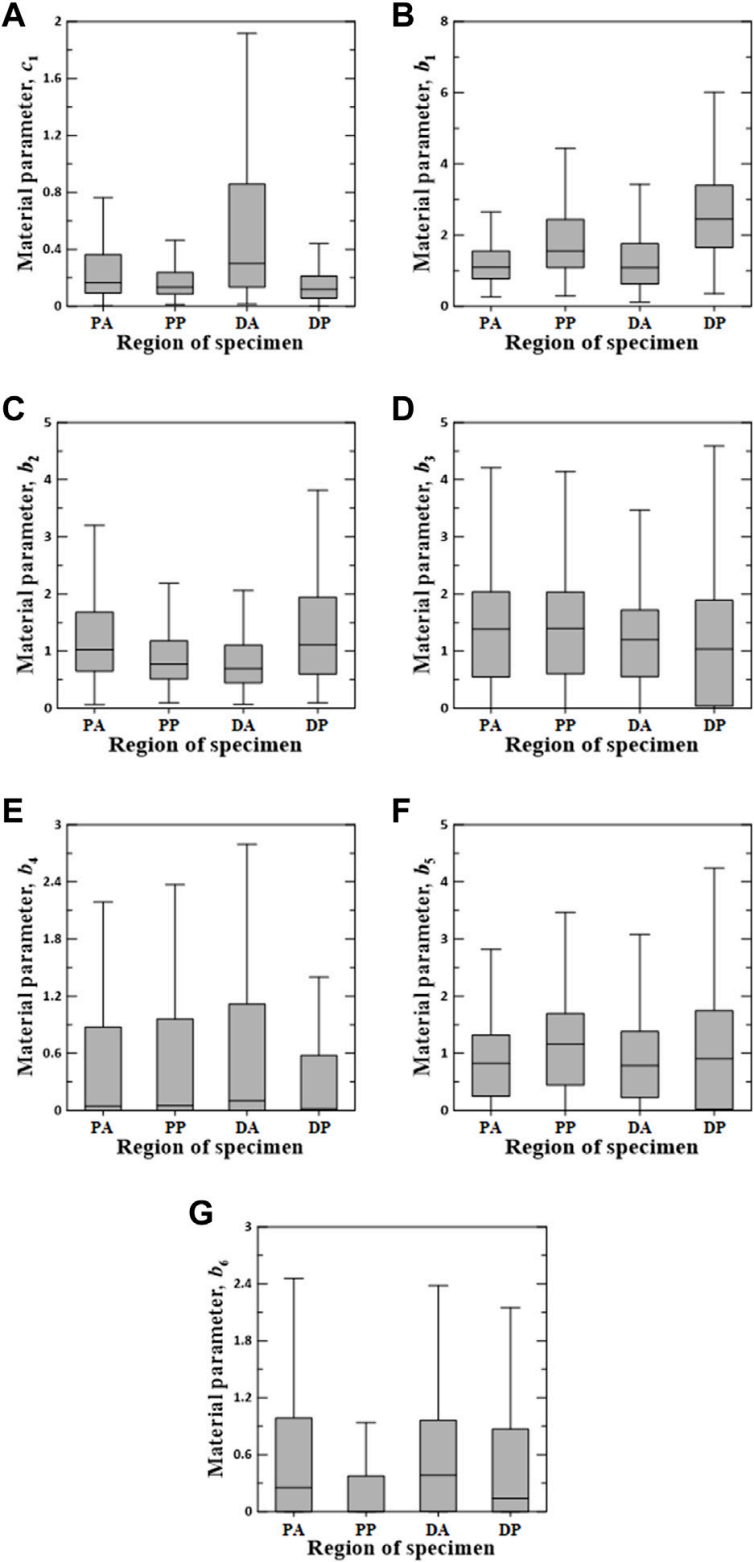
Random value distribution of material parameters estimated using Fung model for PA, PP, DA, and DP specimens. **(A)** c_1_, **(B)** *b*_1_, **(C)** *b*_2_, **(D)** *b*_3_, **(E)** *b*_4_, **(F)** *b*_5_, and **(G)** *b*_6_.

**FIGURE 7:**
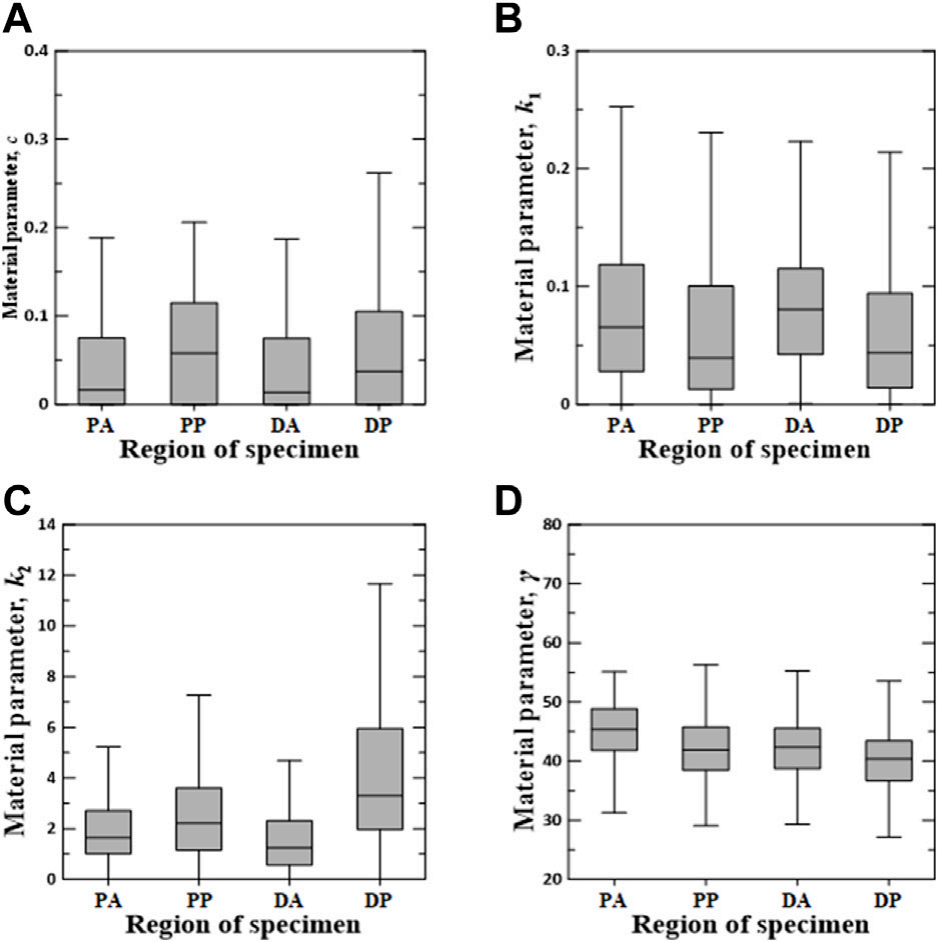
Random value distribution of material parameters estimated using Holzapfel model for PA, PP, DA, and DP specimens. **(A)** *c*, **(B)** *k*_1_, **(C)** *k*_2_, and **(D)** *γ*.

**FIGURE 8:**
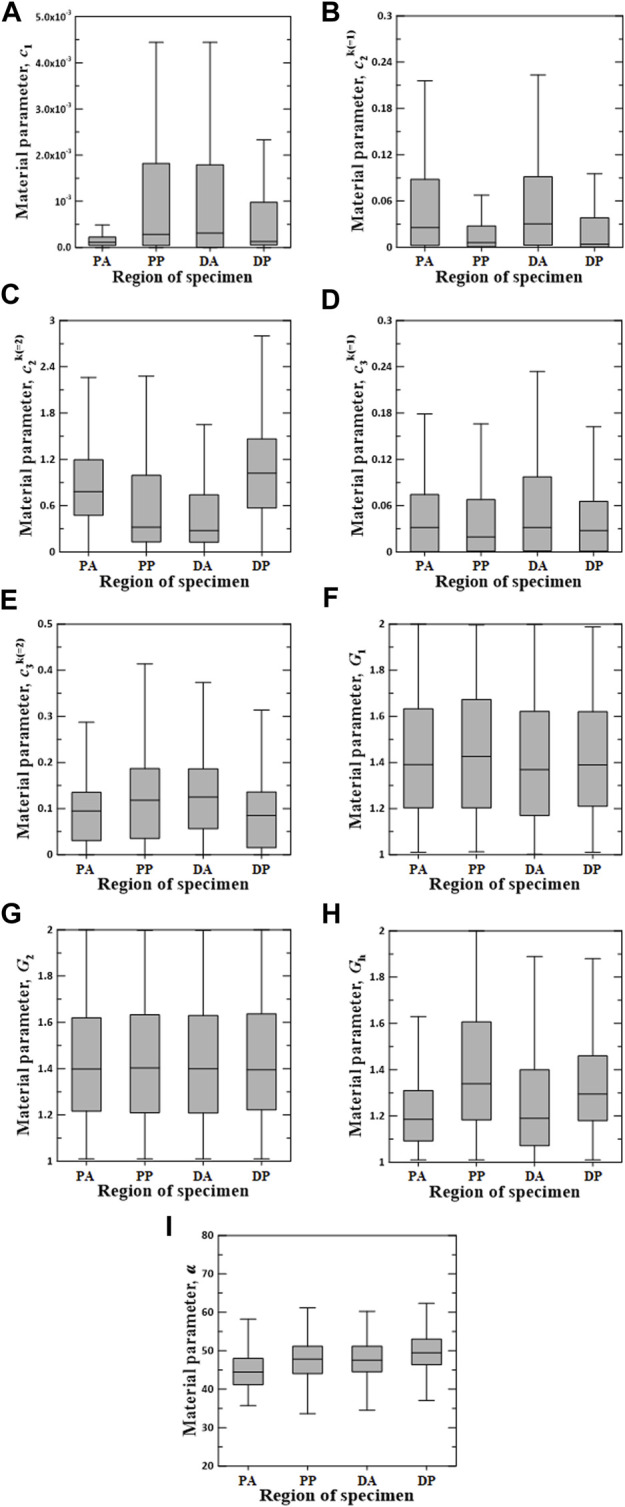
Random value distribution of material parameters estimated using CMM for PA, PP, DA, and DP specimens. **(A)** $c_{1}$ , **(B)** $c_{2}^{1}$ , **(C)** $c_{2}^{2}$ , **(D)** $c_{3}^{1}$ , **(E)** $c_{3}^{2}$ , **(F)** $G_{1}$ , **(G)** $G_{2}$ , **(H)** $G_{h}$ , and **(I)** α .

Figures 6–8 show the Box–Whisker plots for the distribution of the estimated material parameters for three constitutive models based on the experimental data. Material parameters were calculated by sequentially applying pairs of experimental datasets for the circumferential and longitudinal specimens and were estimated without constraints. Subsequently, curve fitting was performed for 9,456 cases [{PAC–PAL (35 × 29 = 1,015 cases), PPC–PPL (25 × 25 = 625 cases), DAC–DAL (26 × 28 = 728 cases), DPC–DPL (28 × 28 = 784 cases)} × three constitutive models]. The minimum, maximum, first quartile (1Q), third quartile (3Q), and median of the estimated material parameters for all three models are listed in the Supplementary Appendix S1.

The values of the material parameters, c_1_, *b*_1_, *b*_2_, *b*_3_, *b*_4_, *b*_5_, and *b*_6_, were obtained using the Fung model, as shown in Figure 6. The material parameters of c_1_ on the DA specimens indicated the widest interquartile ranges (IQRs) and the highest median value of 0.3012. The values of *b*_1_ and *b*_2_ for the DP specimens were the most widely distributed in the IQR, with the highest median values of 2.4510 and 1.1120, respectively. Additionally, material parameters *b*_3_ and *b*_5_ indicated a wide IQR, with slight differences in the median over all test regions. The means ± standard deviations of all median values for parameters *b*_3_ and *b*_5_ were 1.2538 ± 0.1706 and 0.9191 ± 0.1705, respectively. The median values of *b*_4_ and *b*_6_ were relatively low compared with those of the other parameters. In particular, the material parameters of *b*_4_ on the DP specimens and *b*_6_ in the PP region were approximately zero.

The values of material parameters *c*, *k*_1_, *k*_2_, and *γ* obtained using the Holzapfel model are shown in Figure 7. In the regional comparison, the material parameter *c* of the PP specimens exhibited the highest median value of 0.0581. Meanwhile, the material parameter *k*_2_ of the DP specimens indicated the widest IQR, with the highest median value of 3.3020. Material parameters *k*_1_ and *γ* indicated only slight differences over all the test regions.

The values of material parameters $c_{1}$ , $c_{2}^{1}$ , $c_{2}^{2}$ , $c_{3}^{1}$ , $c_{3}^{2}$ , $G_{1}$ , $G_{2}$ , $G_{h}$ , and α for the CMM are shown in Figure 8. In the regional comparison, the values of $c_{1}$ for all test regions were low, and the median values were less than 0.0003. The material parameter $c_{2}^{1}$ of the PA and DA specimens indicated wider IQR distributions with higher mean values compared with those of the other specimens. The material parameter $c_{2}^{2}$ of the DA specimens indicated the narrowest IQR, with the lowest median value of 0.2755, whereas $c_{3}^{1}$ of the DA specimens indicated the widest IQR. Meanwhile, the median value of $c_{2}^{2}$ of the DP specimens was the highest and indicated the widest IQR, whereas that of $c_{3}^{2}$ of the DP specimens was the lowest. The values of $G_{1}$ and $G_{2}$ did not differ significantly among all test regions. The material parameter $G_{h}$ of the PP specimens indicated the widest IQR, with the highest median value of 1.3390. The mean value of α of the PA specimens was slightly lower than those of the others.

### Significant difference of the material parameters

The Student’s t-test for the estimated material parameters was performed to determine the difference among all test regions. It was discovered that material parameters c_1_ and *b*_6_ in the Fung model, *k*_2_ and *γ* in the Holzapfel model, and $c_{2}^{2}$ , $G_{h}$ , and α in the CMM indicated significant differences between proximal and distal regions (PA vs. DA and PP vs. DP), anterior and posterior regions (PA vs. PP and DA vs. DP), and their interactions (PA vs. DP and PP vs. DA) simultaneously. The Student’s t-test shows that the material parameters of the constitutive models depended on the regions of the aortic tissue.

### Statistical correlations

**FIGURE 9:**
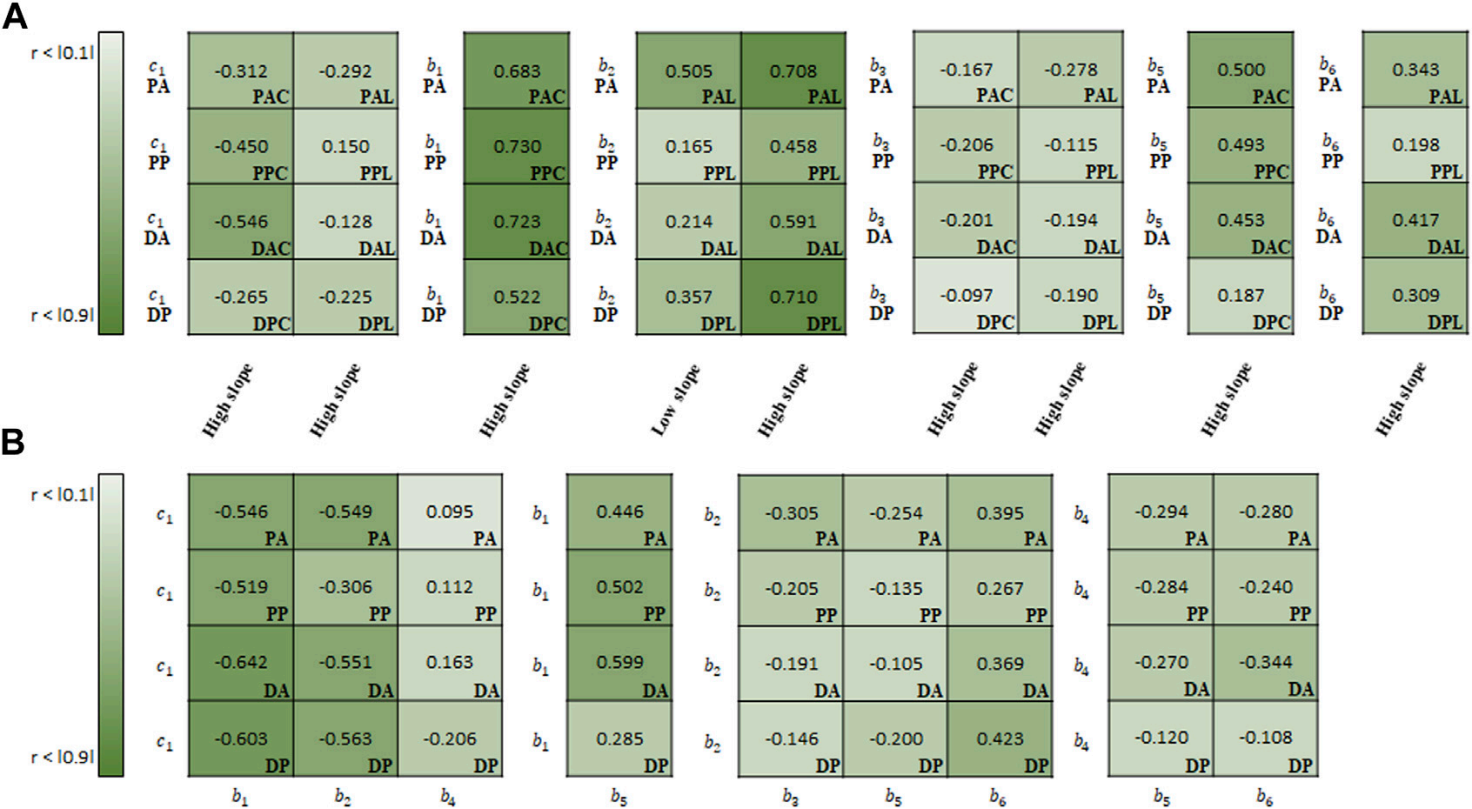
Spearman rank correlation coefficients **(A)** between material parameters of Fung model and experimental results and **(B)** among the material parameters.

**FIGURE 10:**
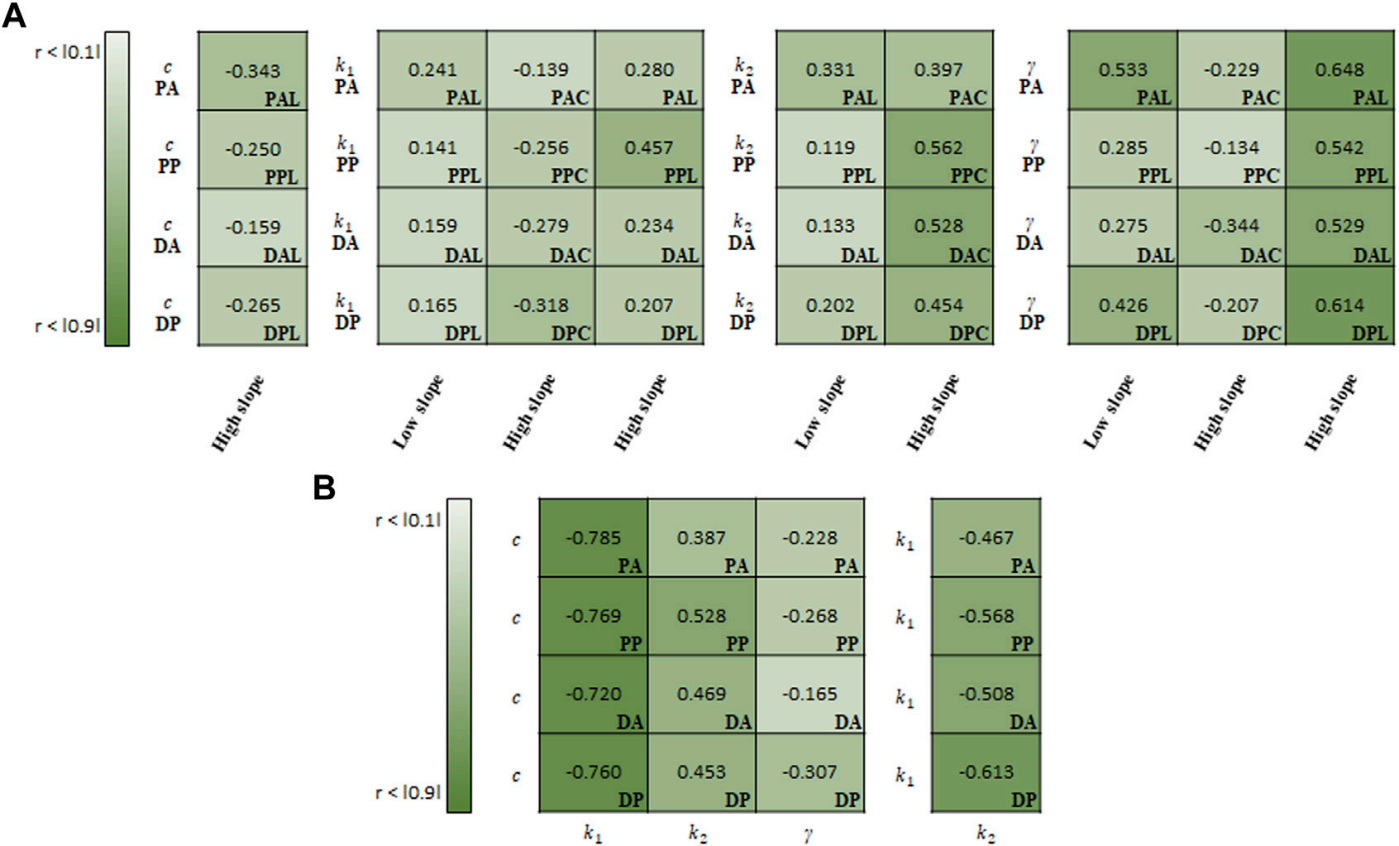
Spearman rank correlation coefficients **(A)** between material parameters of Holzapfel model and experimental results and **(B)** among the material parameters.

**FIGURE 11:**
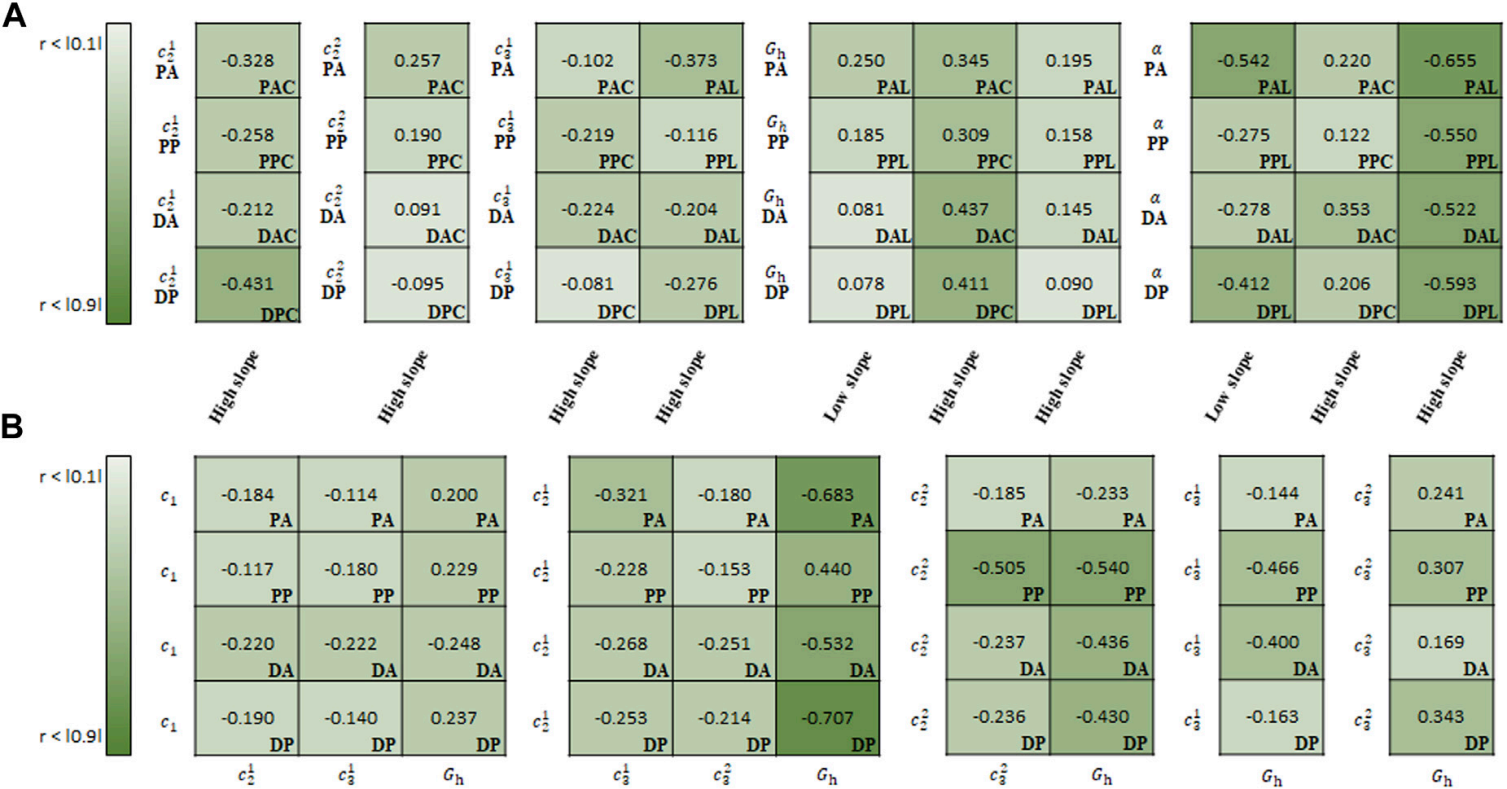
Spearman rank correlation coefficients **(A)** between material parameters of CMM and experimental results and **(B)** among the material parameters.

The estimated material parameters were used sequentially for the correlation study. Spearman rank correlation coefficients (*r*) were computed to quantify the correlation degree between the regional experimental data (i.e., slopes of stress-stretch curves) and material parameters or among the material parameters. Only cases exhibiting a correlation in all test regions are reported, as shown in Figures 9–11. To understand the general trend from the correlation study, the average values of the correlation coefficients over the four test regions (PA, PP, DA, and DP) were obtained.

In the correlation study between the parameters of Fung model and experimental results, *b*_1_ (*r* = 0.665 on average) indicated the strongest correlation with the slope in the high-stiffness regime, hereinafter known as the high slope, in the circumferential specimens, followed by *b*_5_ (0.408) (Figure 9A). Meanwhile, *b*_2_ showed the strong correlation with both high slope (0.617) and low slope (0.310) in the longitudinal specimens.

In the correlation study among the material parameters of the Fung model, *c*_1_ indicated the strongest correlation with *b*_1_ (−0.578), followed by *b*_2_ (−0.492) (Figure 9B). Next, *b*_1_ and *b*_2_ indicated a strong correlation with *b*_5_ (0.458) and *b*_6_ (0.364), respectively.

For the correlation study between the parameters of Holzapfel model and experimental results, *γ* indicated the strongest correlation with the high slope (0.583) and low slope (0.380) in the longitudinal specimens (Figure 10A). Meanwhile, *k*_2_ (0.485) showed the strong correlation with the high slope in the circumferential specimens.

In the correlation study among the material parameters of the Holzapfel model, *c* indicated the strongest correlation with *k*_1_ (−0.759), followed by *k*_2_ (0.459) (Figure 10B). In addition, *k*_1_ was negatively correlated with *k*_2_ (-0.539).

In the correlation study between the parameters of CMM and experimental results, α showed the strongest negative correlation with the high slope (−0. 580) and low slope (−0.377) in the longitudinal specimens (Figure 11A). Meanwhile, $G_{h}$ (0.376) had the strong positive correlation with the high slope in the circumferential specimens, followed by $c_{2}^{1}$ (−0.307) with the negative coefficient.

In the correlation study among the material parameters of the CMM, $c_{2}^{2}$ indicated the strongest correlation with $G_{h}$ (−0.410) (Figure 11B). In the correlation analysis between $c_{2}^{1}$ and $G_{h}$ , the absolute values of the coefficients were the highest, but PP indicated a positive correlation, whereas PA, DA, and DP indicated a negative correlation. The combinations of positive and negative coefficients indicated non-uniform correlations over the test region.

## Discussion

In this study, first of all, a virtual 2D regional dataset is generated by experimental data from uniaxial tensile tests to overcome the limitations of conventional experimental setups to characterize the anisotropic behavior of the artery. We use cross-mapping to determine the material parameters by applying experimental data in both loading directions. The relationship between the physical features (arterial stiffness) and constitutive parameters is determined in different regions. Subsequently, we present the ranges of material parameters and propose an integrative method to characterize the region-dependent material parameters for the nonlinear anisotropic materials.

Kim and Baek (Kim and Baek, 2011) and Kim et al. (Kim et al., 2013) report the spatial variations in the stiffness of the aorta for both circumferential and longitudinal regions *via* the extension-inflation test. In this study, uniaxial tensile tests present consistent significant differences. The circumferential specimen is stiffer than the longitudinal specimen in both the low- and high-stiffness regimes, and the circumferential and longitudinal differences are found among several regions. In particular, the DP exhibits the stiffest slope for each of the circumferential and longitudinal loading directions in both low- and high-stiffness regimes. This is likely due to the non-uniform fiber orientations on the different test regions of the aorta. Various histological and mechanical analyses indicate diverse fiber orientations of the intimal, medial, and adventitial strips (Huang et al., 2016; Niestrawska et al., 2016; Yu et al., 2018; Babici et al., 2020; Concannon et al., 2020; Jadidi et al., 2020; Díaz et al., 2021). Because the DP is the thinnest region of the aorta, the transmural variation in the fiber orientation and the contribution of fibers to the stress–stretch relationship in the DP may differ from those of the other test regions.

Although the material behavior of the aorta is described by a constitutive model, it is not uniquely determined for the best representation. Complex anisotropic materials, such as biological tissues, typically have a single isotropic plane, so that they can be idealized as transversely isotropic in hyperelastic models (O’Shea et al., 2019). In this study, the aorta is assumed to be a transversely isotropic material to simulate the uniaxial tensile test. Although this approach has limitations in ensuring the positive definiteness of the fourth-order stiffness tensor, it provides a stable solution to elasticity problems if a proposed model is calibrated based on experimental data (O’Shea et al., 2019). In addition, in the uniaxial test, the transversely isotropic model to consider only I_4_ invariant shows responses similar to that of the method considering both I_4_ and I_5_ invariants (Feng et al., 2016). In shear deformation, however, both should be considered, as the results depend on whether I_5_ invariant is considered.

In this study, three constitutive models, i.e., the Fung model, Holzapfel model, and CMM, are considered with this methodology to describe the material responses in the uniaxial test. All three models present good fits with the stress–stretch curves obtained from the uniaxial test. The Fung model has the advantage of fitting curves with a large variation, whereas the Holzapfel model and CMM have the significant advantage of considering vascular constituents such as elastin and collagen. In the CMM, the highest number of material parameters is used, including the pre-stretch value in the homeostatic state. Nevertheless, it presents a substantial advantage in that it can accurately describe nonlinear behavior of the aorta, particularly in the high-stiffness regime. To improve the computational efficiency of the CMM composed of many parameters, the estimation method is classified into two steps in a previous study (Seyedsalehi et al., 2015). However, unlike the previous study, the method used in this study involves integrating material parameters for the two loading directions into the virtual biaxial behavior. No significant difference in the computational efficiency is observed between the two methods regardless of integration.

This study shows that the material parameters of each constitutive model can express the mechanical behavior of the regional aortic tissue. There are significant differences in the material parameters with respect to the regions. For example, the material parameter *γ* in the Holzapfel model and the material parameter α in the CMM exhibit the fiber orientation of the artery depending on the test regions. It is, however, important to note that, although *γ* and α represent the fiber orientations, the single parameter alone cannot have much weight on interpreting the physical meaning due to its inter-correlation with other parameters. Evidently, *γ* in the Holzapfel model indicated the highest mean for the PA, whereas α in the CMM indicated the highest mean for the DP.

In addition to regional differences, strong correlations are presented between regional aortic behavior and material parameters or among material parameters for the same test region. As shown in Figures 9–11, a higher absolute value of the correlation coefficient indicates a stronger correlation. In the Fung model, the material parameters c_1_, *b*_1_, *b*_2_, *b*_3_, *b*_5_, and *b*_6_ correlate with the slope of the high-stiffness regime. Although the material parameters of the Fung model provide a description of the stress–stretch relationship phenomenologically, they are distinct with respect to the regions and regimes when describing the mechanical behavior with the physical characteristics and slopes. In particular, the material parameters *b*_1_ and *b*_2_ have a direct effect on the simulating the stiffness of the materials in each loading direction, because they are multiplied by the stretch in each loading direction. The ratio of *b*_1_/*b*_2_ can be used to characterize the anisotropic behavior of the materials. In this study, this ratio may give a better way to represent the behavior of materials in the correlation study between the ratio and stiffness regime. It ranges from −0.473 to −0.171 among the slopes in the low-stiffness regime for longitudinal specimens, and from 0.298 to 0.503/from −0.645 to −0.473 among the slopes in the high-stiffness regime for circumferential/longitudinal specimens, respectively. In the Holzapfel model, all parameters correlate with the slope in the high-stiffness regime, while the parameters *k*_1_, *k*_2_, and *γ* correlate with the slope in the low-stiffness regime for the longitudinal specimens. In addition, the parameter *c* is inter-correlated with all other parameters, and particularly has the strongest negative correlation with *k*_1_. Finally, in the CMM, the parameters $c_{2}^{1}$ , $c_{2}^{2}$ , $c_{3}^{1}$ , $G_{h}$ , and α correlate with the slope in the high-stiffness regime, while $G_{h}$ and α show the correlation with the slope in the low-stiffness regime for the longitudinal specimens. In particular, $G_{h}$ has the greatest inter-correlation with others, *c*_1_, $c_{2}^{1}$ , $c_{2}^{2}$ , $c_{3}^{1}$ , and $c_{3}^{2}$ with $c_{2}^{1}$ showing the strongest correlation. In this correlation study, these findings support the assumption that the three constitutive models can simulate the region-dependent mechanical behavior of the aorta in both low- and high-stiffness regimes.

There are limitations of this study. For the experimental protocol, the deformation of aortic segments is assumed to be uniform, whereas the sample might not deform uniformly since the blood vessel is a composite material. However, the specimens are small, and their spatial variations are generally neglected in the uniaxial tensile test. Especially, the stretch is defined by the change in distance between two microspheres because it gives an accurate estimation of local deformation at the central region, instead of measuring the grip movement in the uniaxial test. Nonetheless, the specimens are not necessarily ruptured in the central region between the two microspheres so the uniformity assumption cannot apply to the rupture behavior. To further investigate the nonuniform deformation, adding more markers to the sample or utilizing the digital image correlation technique may be an alternative.

The statistical analysis performed in this study confirms that the statistical significance of key parameters is important in differentiating the regional mechanical behavior of the aorta. However, the constitutive models used in this study are nonlinear and, as shown in the correlation studies, the physical characteristics (e.g., stiffness in high-stiffness regime) are highly correlated with the material parameters of each model. Analyzing such multidimensional data (regions, loading directions, low- and high-stiffness regimes, constitutive models and material parameters, and enhanced experimental measurements) requires further studies using a machine learning approach to predict the regional mechanical behavior of the blood vessel for the virtual aorta.

Due to the complexity between the clinical features and model parameters, the number of model parameters is reduced to estimate the physical features within the model. A decrease in the number of independent parameters may be more advantageous, based on overall nonlinear sensitivity analysis and model uncertainty quantification. For example, a multidimensional virtual dataset can quantify model uncertainties through correlation studies. A linear regression model with Bayesian inference approaches can be used to describe significant material parameters using backward elimination (Akkoyun et al., 2020). Alternatively, the Gaussian mixture model is based on an unsupervised machine learning approach involving an uncertainty measure (probability) (Paalanen et al., 2006), which determines how much the material parameters are associated with a specific region by considering the mean and covariance of each cluster and mixing probability. Therefore, the combination of material parameters can improve the prediction capability of the model with respect to the different regions and the selection of a constitutive model, appropriately characterizing the regional mechanical behavior of the blood vessel based on a machine learning approach.

In summary, this study presents the region-dependent mechanical characterization of the porcine thoracic aorta by generating virtual 2D datasets from the uniaxial test using three constitutive models. Physics-based modeling is essential to associate with physical and clinical features. The development of computational models can further promote the era of “virtual human” or “virtual aorta.” However, the development of specific models is hampered by the scarcity of material test data for individual patients, which are relevant to clinical setups. In particular, tissue mechanical tests of young human aortic specimens are rarely available, even if they exist. The virtual biaxial data presented herein may serve as an alternative to material behavior in young human aortas.

## Data Availability

The original contributions presented in the study are included in the article/Supplementary Material, further inquiries can be directed to the corresponding author.
