# Supplementary material for: Region-dependent mechanical characterization of porcine thoracic aorta with a one-to-many correspondence method to create virtual datasets using uniaxial tensile tests
Source: Front Bioeng Biotechnol. 2022 Oct 11;10:937326. doi: 10.3389/fbioe.2022.937326 (PMC9595283; doi:10.3389/fbioe.2022.937326)
Supplement: Supplementary file 1 [file Table1.docx]

**Table 1.** Values of material parameters in Fung model

| **Fung model** | **c_1_** | ***b*_1_** | ***b*_2_** | ***b*_3_** | ***b*_4_** |
| --- | --- | --- | --- | --- | --- |
| minimum | 4.40E-03 (PA)  0.01 (PP)  0.01 (DA)  5.96E-04 (DP) | 0.26 (PA)  0.29 (PP)  0.12 (DA)  0.35 (DP) | 0.07 (PA)  0.10 (PP)  0.07 (DA)  0.10 (DP) | 6.77E-10 (PA)  8.35E-10 (PP)  1.01E-09 (DA)  1.21E-09 (DP) | 1.73E-11 (PA)  6.93E-10 (PP)  1.23E-11 (DA)  5.57E-11 (DP) |
| 1Q | 0.09 (PA)  0.09 (PP)  0.13 (DA)  0.06 (DP) | 0.77 (PA)  1.09 (PP)  0.62 (DA)  1.65 (DP) | 0.65 (PA)  0.51 (PP)  0.44 (DA)  0.60 (DP) | 0.55 (PA)  0.60 (PP)  0.55 (DA)  0.04 (DP) | 5.00E-06 (PA)  3.53E-06 (PP)  4.95E-06 (DA)  1.87E-06 (DP) |
| median | 0.17 (PA)  0.13 (PP)  0.30 (DA)  0.12 (DP) | 1.10 (PA)  1.56 (PP)  1.09 (DA)  2.45 (DP) | 1.03 (PA)  0.78 (PP)  0.70 (DA)  1.11 (DP) | 1.38 (PA)  1.40 (PP)  1.20 (DA)  1.04 (DP) | 0.04 (PA)  0.05 (PP)  0.10 (DA)  0.02 (DP) |
| 3Q | 0.36 (PA)  0.24 (PP)  0.86 (DA)  0.21 (DP) | 1.55 (PA)  2.44 (PP)  1.76 (DA)  3.40 (DP) | 1.68 (PA)  1.18 (PP)  1.11 (DA)  1.94 (DP) | 2.04 (PA)  2.04 (PP)  1.72 (DA)  1.89 (DP) | 0.88 (PA)  0.96 (PP)  1.12 (DA)  0.58 (DP) |
| maximum | 0.76 (PA)  0.46 (PP)  1.92 (DA)  0.44 (DP) | 2.65 (PA)  4.44 (PP)  3.43 (DA)  6.02 (DP) | 3.20 (PA)  2.18 (PP)  2.06 (DA)  3.81 (DP) | 4.21 (PA)  4.14 (PP)  3.47 (DA)  4.59 (DP) | 2.19 (PA)  2.37 (PP)  2.79 (DA)  1.40 (DP) |
| **Fung model** | ***b*_5_** | ***b*_6_** |  |  |  |
| minimum | 1.66E-11 (PA)  7.12E-10 (PP)  1.77E-10 (DA)  2.79E-10 (DP) | 2.22E-10 (PA)  2.19E-11 (PP)  1.93E-10 (DA)  3.09E-11 (DP) |  |  |  |
| 1Q | 0.25 (PA)  0.44 (PP)  0.23 (DA)  0.02 (DP) | 6.36E-05 (PA)  1.10E-06 (PP)  7.00E-04 (DA)  1.70E-05 (DP) |  |  |  |
| median | 0.82 (PA)  1.16 (PP)  0.79 (DA)  0.90 (DP) | 0.25 (PA)  2.80E-03 (PP)  7.00E-04 (DA)  0.14 (DP) |  |  |  |
| 3Q | 1.32 (PA)  1.70 (PP)  1.38 (DA)  1.75 (DP) | 0.99 (PA)  0.38 (PP)  0.96 (DA)  0.87 (DP) |  |  |  |
| maximum | 2.82 (PA)  3.47 (PP)  3.08 (DA)  4.24 (DP) | 2.46 (PA)  0.93 (PP)  2.38 (DA)  2.15 (DP) |  |  |  |
