# Supplementary material for: Region-dependent mechanical characterization of porcine thoracic aorta with a one-to-many correspondence method to create virtual datasets using uniaxial tensile tests
Source: Front Bioeng Biotechnol. 2022 Oct 11;10:937326. doi: 10.3389/fbioe.2022.937326 (PMC9595283; doi:10.3389/fbioe.2022.937326)
Supplement: Supplementary file 2 [file Table2.docx]

**Table 2.** Values of material parameters in Holzapfel model

| **Holzapfel model** | ***c*** | ***k*_1_** | ***k*_2_** | ***γ*** |
| --- | --- | --- | --- | --- |
| minimum | 1.50E-11 (PA)  1.19E-11 (PP)  1.91E-11 (DA)  1.19E-11 (DP) | 1.15E-05 (PA)  1.59E-05 (PP)  4.21E-04 (DA)  1.16E-04 (DP) | 1.07E-08 (PA)  4.57E-09 (PP)  4.24E-06 (DA)  1.17E-07 (DP) | 31° (PA)  29° (PP)  29° (DA)  27° (DP) |
| 1Q | 5.15E-08 (PA)  1.48E-07 (PP)  4.73E-07 (DA)  4.17E-06 (DP) | 0.03 (PA)  0.01 (PP)  0.04 (DA)  0.01 (DP) | 1.01 (PA)  1.15 (PP)  0.57 (DA)  1.96 (DP) | 41° (PA)  38° (PP)  39° (DA)  37° (DP) |
| median | 0.02 (PA)  0.06 (PP)  0.01 (DA)  0.04 (DP) | 0.07 (PA)  0.04 (PP)  0.08 (DA)  0.04 (DP) | 1.65 (PA)  2.21 (PP)  1.24 (DA)  3.30 (DP) | 45° (PA)  42° (PP)  42° (DA)  40° (DP) |
| 3Q | 0.08 (PA)  0.11 (PP)  0.07 (DA)  0.10 (DP) | 0.12 (PA)  0.10 (PP)  0.12 (DA)  0.09 (DP) | 2.71 (PA)  3.61 (PP)  2.31 (DA)  5.95 (DP) | 49° (PA)  46° (PP)  46° (DA)  43° (DP) |
| maximum | 0.19 (PA)  0.21 (PP)  0.19 (DA)  0.26 (DP) | 0.25 (PA)  0.23 (PP)  0.22 (DA)  0.21 (DP) | 5.23 (PA)  7.27 (PP)  4.69 (DA)  11.65 (DP) | 55° (PA)  56° (PP)  55° (DA)  54° (DP) |
