# Supplementary material for: Region-dependent mechanical characterization of porcine thoracic aorta with a one-to-many correspondence method to create virtual datasets using uniaxial tensile tests
Source: Front Bioeng Biotechnol. 2022 Oct 11;10:937326. doi: 10.3389/fbioe.2022.937326 (PMC9595283; doi:10.3389/fbioe.2022.937326)
Supplement: Supplementary file 3 [file Table3.docx]

**Table 3.** Values of material parameters in CMM

| **CMM** | $\boldsymbol{c}_{\mathbf{1}}$ | $\boldsymbol{c}_{\boldsymbol{2}}^{\boldsymbol{1}}$ | $\boldsymbol{c}_{\boldsymbol{2}}^{\boldsymbol{2}}$ | $\boldsymbol{c}_{\boldsymbol{3}}^{\boldsymbol{1}}$ | $\boldsymbol{c}_{\boldsymbol{3}}^{\boldsymbol{2}}$ |
| --- | --- | --- | --- | --- | --- |
| minimum | 2.02E-14 (PA)  1.33E-15 (PP)  2.25E-13 (DA)  3.88E-14 (DP) | 1.08E-11 (PA)  1.57E-12 (PP)  1.23E-11 (DA)  7.54E-11 (DP) | 2.12E-11 (PA)  6.76E-11 (PP)  2.89E-12 (DA)  6.65E-11 (DP) | 5.38E-11 (PA)  6.24E-14 (PP)  3.56E-11 (DA)  8.66E-12 (DP) | 6.59E-11 (PA)  1.58E-12 (PP)  1.72E-11 (DA)  1.25E-10 (DP) |
| 1Q | 4.52E-05 (PA)  4.12E-05 (PP)  1.19E-06 (DA)  5.64E-05 (DP) | 2.40E-03 (PA)  6.00E-04 (PP)  2.70E-03 (DA)  4.00E-04 (DP) | 0.47 (PA)  0.13 (PP)  0.13 (DA)  0.57 (DP) | 5.00E-04 (PA)  9.00E-04 (PP)  1.60E-03 (DA)  1.00E-03 (DP) | 0.03 (PA)  0.04 (PP)  0.06 (DA)  0.02 (DP) |
| median | 1.00E-04 (PA)  3.00E-04 (PP)  3.00E-04 (DA)  1.00E-04 (DP) | 0.03 (PA)  6.00E-03 (PP)  0.03 (DA)  4.20E-03 (DP) | 0.78 (PA)  0.32 (PP)  0.28 (DA)  1.02 (DP) | 0.03 (PA)  0.02 (PP)  0.03 (DA)  0.03 (DP) | 0.09 (PA)  0.12 (PP)  0.12 (DA)  0.08 (DP) |
| 3Q | 2.00E-04 (PA)  1.80E-03 (PP)  1.80E-03 (DA)  1.00E-03 (DP) | 0.09 (PA)  0.03 (PP)  0.09 (DA)  0.04 (DP) | 1.20 (PA)  0.99 (PP)  0.74 (DA)  1.47 (DP) | 0.07 (PA)  0.07 (PP)  0.10 (DA)  0.07 (DP) | 0.14 (PA)  0.19 (PP)  0.19 (DA)  0.14 (DP) |
| maximum | 5.00E-04 (PA)  4.40E-03 (PP)  4.40E-03 (DA)  2.30E-03 (DP) | 0.22 (PA)  0.07 (PP)  0.22 (DA)  0.10 (DP) | 2.26 (PA)  2.28 (PP)  1.65 (DA)  2.80 (DP) | 0.18 (PA)  0.17 (PP)  0.23 (DA)  0.16 (DP) | 0.29 (PA)  0.41 (PP)  0.37 (DA)  0.31 (DP) |
| **CMM** | $\boldsymbol{G}_{\mathbf{1}}$ | $\boldsymbol{G}_{\mathbf{2}}$ | $\boldsymbol{G}_{\mathbf{h}}$ | $\alpha$ |  |
| minimum | 1.01 (PA)  1.01 (PP)  1.00 (DA)  1.01 (DP) | 1.01 (PA)  1.01 (PP)  1.01 (DA)  1.01 (DP) | 1.01 (PA)  1.01 (PP)  1.00 (DA)  1.01 (DP) | 36° (PA)  34° (PP)  35° (DA)  37° (DP) |  |
| 1Q | 1.20 (PA)  1.20 (PP)  1.17 (DA)  1.21 (DP) | 1.22 (PA)  1.21 (PP)  1.21 (DA)  1.22 (DP) | 1.09 (PA)  1.18 (PP)  1.07 (DA)  1.18 (DP) | 41° (PA)  44° (PP)  45° (DA)  46° (DP) |  |
| median | 1.39 (PA)  1.43 (PP)  1.37 (DA)  1.39 (DP) | 1.40 (PA)  1.40 (PP)  1.40 (DA)  1.40 (DP) | 1.19 (PA)  1.34 (PP)  1.19 (DA)  1.30 (DP) | 45° (PA)  48° (PP)  48° (DA)  50° (DP) |  |
| 3Q | 1.63 (PA)  1.67 (PP)  1.62 (DA)  1.62 (DP) | 1.62 (PA)  1.63 (PP)  1.63 (DA)  1.64 (DP) | 1.31 (PA)  1.61 (PP)  1.40 (DA)  1.46 (DP) | 48° (PA)  51° (PP)  51° (DA)  53° (DP) |  |
| maximum | 2.00 (PA)  2.00 (PP)  2.00 (DA)  1.99 (DP) | 2.00 (PA)  2.00 (PP)  2.00 (DA)  2.00 (DP) | 1.63 (PA)  2.00 (PP)  1.89 (DA)  1.88 (DP) | 58° (PA)  61° (PP)  60° (DA)  62° (DP) |  |
